# MicroRNAs as the pivotal regulators of cisplatin resistance in head and neck cancers

**DOI:** 10.1186/s12935-023-03010-9

**Published:** 2023-08-16

**Authors:** Faezeh Tolue Ghasaban, Amirhosein Maharati, Amir Sadra Zangouei, Alireza Zangooie, Meysam Moghbeli

**Affiliations:** 1 Department of Medical Genetics and Molecular Medicine, School of Medicine, Mashhad University of Medical Sciences, Mashhad, Iran; 2 Medical Genetics Research Center, Mashhad University of Medical Sciences, Mashhad, Iran; 3 Student Research Committee, Faculty of Medicine, Mashhad University of Medical Sciences, Mashhad, Iran; 4 Cellular and Molecular Research Center, Birjand University of Medical Sciences, Birjand, Iran; 5 Student research committee, Birjand University of Medical Sciences, Birjand, Iran

**Keywords:** Head and neck cancer, MicroRNAs, Cisplatin, Drug resistance, Diagnosis, Panel marker

## Abstract

Although, there is a high rate of good prognosis in early stage head and neck tumors, about half of these tumors are detected in advanced stages with poor prognosis. A combination of chemotherapy, radiotherapy, and surgery is the treatment option in head and neck cancer (HNC) patients. Although, cisplatin (CDDP) as the first-line drug has a significant role in the treatment of HNC patients, CDDP resistance can be observed in a large number of these patients. Therefore, identification of the molecular mechanisms involved in CDDP resistance can help to reduce the side effects and also provides a better therapeutic management. MicroRNAs (miRNAs) as the post-transcriptional regulators play an important role in drug resistance. Therefore, in the present review we investigated the role of miRNAs in CDDP response of head and neck tumors. It has been reported that the miRNAs exerted their roles in CDDP response by regulation of signaling pathways such as WNT, NOTCH, PI3K/AKT, TGF-β, and NF-kB as well as apoptosis, autophagy, and EMT process. The present review paves the way to suggest a non-invasive miRNA based panel marker for the prediction of CDDP response among HNC patients. Therefore, such diagnostic miRNA based panel marker reduces the CDDP side effects and improves the clinical outcomes of these patients following an efficient therapeutic management.

## Background

Head and neck cancers (HNCs) are a heterogeneous group of malignancies originating from the epithelium of the upper aero-digestive tracts [1, 2]. HNCs are the sixth most frequent malignancies globally that account for 5.3% of all cancers [3]. HNC incidence has been growing during the past decade in which almost 880,000 newly diagnosed cases and 450,000 mortalities have been reported annually worldwide [4]. Lip and oral cavity cancers are the most common types of HNC with 377,713 new cases and 177,757 deaths, followed by larynx cancer with 184,615 new cases and 99,840 deaths in 2020 [5, 6]. Surgery and chemo radiotherapy are the most common therapeutic methods in HNC patients. Surgery or radiotherapy alone has been shown to be sufficient for treating and enhancing survival rates of nearly one-third of the early-stage HNC cases. However, most of the advanced-stage HNC cases require chemotherapy alone or in combination with radiotherapy, which have favorable results in improving the patient’s survivals [7, 8]. Head and neck squamous cell carcinoma (HNSCC) is the most frequent pathological HNC type [9]. The locally advanced HNSCC is a poor prognostic cancer with the 5-year survival rate of 40–60%. Metastatic HNSCC or loco regional recurrence of tumor cells has a poor response to the surgery or radiation therapy [10, 11]. Cisplatin (CDDP), docetaxel, paclitaxel, and 5-fluorouracil (5-FU) are the first line chemotherapeutic regimens that are typically used for HNC patients [12, 13]. Cisplatin exerts its cytotoxic effects via DNA adducts which results in induction of DNA damage response and apoptosis [14]. It can also interact with different cytoplasmic molecules resulting in the formation of reactive oxygen species which further promotes DNA damage [15]. Cisplatin has a wide spectrum of the side effects in HNC patients including dermatitis, dysphagia, kidney injury, anemia, and hearing loss [16, 17]. Chemo-radiotherapy is the main clinical approach for nasopharyngeal carcinoma (NPC). However, despite the considerable advances in treatment methods, the 5-year survival rate remains significantly low because of the distant metastasis and drug resistance. Cisplatin (CDDP) is used as the first-line drug for the NPC treatment. Due to CDDP resistance and dose-related cytotoxicity, which are key barriers for the successful NPC treatment, the efficiency of CDDP-based chemotherapy is limited [18]. Oral squamous cell carcinoma (OSCC) is the most common HNC that contains almost 90% of oral cancers [19]. A combination of the surgery, chemotherapy, and radiotherapy is recommended as the common treatment option for the OSCC [20]. Cisplatin is the first-line therapeutic method in OSCC [21, 22]. However, cisplatin resistance is commonly observed among the OSCC patients with tumor relapse who have poor clinical outcomes [23]. Cisplatin is widely used to treat advanced-stage laryngeal carcinoma, however there is a poor prognosis due to cisplatin resistance among laryngeal cancer patients [24, 25]. Neoadjuvant systemic therapy is one of the therapeutic options to reduce death rate in advanced tongue cancer. On the other hand, chemotherapy has some limitations due to the intrinsic or acquired drug-resistance. A considerable number of tongue cancers are found to be resistant to chemotherapy, which leads to a more aggressive phenotype and poor prognosis [26, 27].

Regarding the dose-related cytotoxicity and side effects of CDDP in normal tissues and organs, it is necessary to predict the CDDP response in head and neck tumors. Therefore, the molecular mechanisms of CDDP resistance should be clarified in these tumors to reduce the side effects and introduce the most efficient therapeutic methods. CDDP resistance can be associated with a variety of cellular processes, including drug efflux, enhanced DNA repair, and stimulation of drug-detoxifying systems [28]. Mismatch repair (MMR) is a DNA repair mechanism involved in single-strand DNA (ssDNA) errors repair. MMR pathway is accomplished by MutSα (MSH2-MSH6) and MutSβ (MSH2-MSH3) [29]. It has been shown that aberrant MMR can be associated with CDDP resistance and poor prognosis. MMR has a key role to maintain CDDP interstrand DNA cross-links that promotes cellular sensitivity. MSH2-MSH6 complex is required for the CDDP sensitivity [30]. MicroRNAs (miRNAs) function as the key post-transcriptional regulators through the inhibition of mRNA translation or mRNA degradation [28]. About one third of human miRNAs are organized in clusters that can be transcribed in a single transcriptional unit. Although, majority of the miRNAs are intronic, a minor group of miRNAs are located in repetitive sequences. The miRNA biogenesis begins with the pri-miRNA transcription by RNA polymerase II that can be processed to pre-miRNA by Drosha/DGCR8 complex. Subsequently, pre-miRNA is exported to the cytoplasm to convert to the mature duplex miRNA by Dicer [31]. MiRNAs have pivotal roles in regulation of cell proliferation, cell death, migration, and drug resistance as oncogenes or tumor suppressors [14, 32]. They have higher stabilities in body fluids compared with mRNAs enabling to suggest them as the non-invasive diagnostic biomarkers for cancer patients [33]. Since, miRNAs have key roles in regulation of CDDP response in head and neck tumors [34, 35]; in the present review we discussed the cell and molecular mechanisms by which miRNAs affect the CDDP response in these tumors (Table 1).

**Table 1:** All of the miRNAs associated with Cisplatin (CDDP) resistance in head and neck tumors

STUDY	YEAR	TUMOR TYPE	GENE	TARGET	SAMPLES	RESULTS
Song (35)	2021	LSCC	miR-497-5p	SEPT2	38 patients Hep2, TU212, TU686, SCC-2 and 16HBE cell lines	Increased CDDP sensitivity
Lin (46)	2016	OSCC	miR-203	PIK3CA	10 patients Tca8113 cell line	Increased CDDP Sensitivity
Wang (50)	2017	NPC	ANRIL	let-7a	35 patients CNE1, CNE2, S18, HONE1, and 5–8 F cell lines	Increased CDDP resistance
Lin (51)	2020	NPC	miR-454-3p	c-Met	96 patients C666-1 and HNE1 cell lines	Increased CDDP sensitivity
Zheng (53)	2015	TSCC	miR-24	PTEN	79 patients NHOK, UMI, UM2, Cal27, SCCI, SCC2, SCC9, SCC15 and SCC25 cell lines	Increased CDDP resistance
Sheng (54)	2022	HNSCC	miR-21	PTEN	30 patients UMSCC-1, UMSCC-10 A, UMSCC-22B, Cal33, UPCI-4B, UPCI-15B, 1483, and 686LN cell lines	Increased CDDP resistance
Zhen (56)	2017	NPC	miR-374a	CCND1	239 patients SUNE1, 5 − 8 F, shPDCD4-SUNE1, shPDCD4-HONE1 and PDCD4-overexpressed 5 − 8 F cell lines	Increased CDDP sensitivity
Liu (57)	2017	OSCC	miR-21	PTEN and PDCD4	HSC-3 and SCC-9 cell lines	Increased CDDP resistance
Shi (62)	2016	NPC	miR-26b	JAG1	66 patients CNE2, HNE1, HNE1/DDP and CNE2/DDP cell lines	Increased CDDP sensitivity
Yuan (66)	2018	laryngeal carcinoma	miR-320a	RBPJ	24 patients HEp-2 cell line	Increased CDDP sensitivity
Zeng (72)	2021	OSCC	miR-4786-3p	SELENBP1	Cal27, HSC3, SCC25, HOK and SCC9 cell lines	Increased CDDP resistance
Zhuang (75)	2017	oral cancer	miR-218	PPP2R5A	61 patients UM1, UM2, Cal27, MD1386Ln and Tca8113 cell lines	Increased CDDP resistance
Wang (77)	2017	NPC	miR-183	MTA1	29 patients C666-1, CNE1, CNE2, HONE1, and 5-8 F cell lines	Increased CDDP sensitivity.
Shibata (78)	2022	HNSCC	miR-766-3p	NR3C2	12 patients CAL27 and FaDu cell lines	Increased CDDP resistance
Yuan (80)	2019	laryngeal carcinoma	miR-425-5p	PTCH1	24 patients Hep-2 Hep-2/R Cell lines	Increased CDDP sensitivity
Bissey (96)	2020	NPC	miR-34c	SOX4	246 patients C666–1, NP69, NP460, and HEK 293T cell lines	Increased CDDP sensitivity
Chen (97)	2020	OSCC	miR-132	TGF-β1	37 patients SCC-9 and CAL-27 cell lines	Increased CDDP sensitivity
Gu (100)	2018	TSCC	miR-22	KAT6B	28 patients CAL27, SCC9, and HCT 116 cell lines	Increased CDDP sensitivity
Lin (102)	2021	NPC	miR-515-5p	IL-25	138 patients HK-1 and CNE-1 cell lines	Increased CDDP sensitivity
Liu (109)	2015	ACC	miR-101-3p	Pim-1	30 patients SACC-LM and SACC-83 cell lines	Increased CDDP sensitivity
Wang (110)	2019	OSCC	miR-214-3p	PIM-1	31 patients NHOK, OSCC, TSCCA, CAL-27, SCC-9, and Tca8113 cell lines	Increased CDDP sensitivity
Tian (116)	2017	laryngeal cancer	miR-26b	ATF2	Hep-2 and Hep-2/R cell lines	Increased CDDP sensitivity.
Fan (123)	2015	TSCC	miR-483-5p	FIS1	108 patients CAL27 and SCC9 cell lines	Increased CDDP resistance
Chen (130)	2015	NPC	miR-125a and miR-125b	p53 mRNA	10 patients TW03, CNE-1, CNE-2 and NP69	Increased CDDP resistance
Wang (134)	2020	OSCC	miR-421	MEIS2	45 patients NHOK, CAL-27, Tca8113, SCC-9, and TSCCA	Increased CDDP sensitivity
Liu (138)	2016	laryngeal carcinoma	miR-125a	HAX-1	30 patients Hep-2 cell line	Increased CDDP sensitivity.
Lin (140)	2020	LSCC	miR-936	GPR78	25 patients Hep-2, 16HBE, HEK293T and KB-3-1 cell lines	Increased CDDP sensitivity
Chen (148)	2021	LSCC	miR-107	HMGB1	30 patients	Increased CDDP sensitivity
Zhao (149)	2020	NPC	miR-1278	ATG2B	90 patients CNE-1, CNE-2, C666-1, 5–8 F and HONE-1 cell lines	Increased CDDP sensitivity
Feng (150)	2021	laryngeal carcinoma	miR-376a	ATG2A	30 patients SNU46 and M4E cell lines	Increased CDDP sensitivity
Hao (155)	2020	NPC	miR-205	HER3	CNE1, CNE2, SUNE1 and HK1 cell lines	Increased CDDP sensitivity
Zhang (160)	2019	NPC	miR-205-5p	PTEN	HNE1 and HNE1/DDP cell lines	Increased CDDP resistance
Peng (162)	2015	TSCC	miR-23a	Twist1	SCC-4 and Tca8113 cell lines	Increased CDDP resistance
Wang (163)	2017	TSCC	miR-15b	TRIM14	SCC25 and SCC25-res cell lines	Increased CDDP sensitivity
Li (167)	2021	NPC	miR-98	PBX3	40 patients NP-69 and 5-8 F cell lines	Increased CDDP sensitivity
Sun (170)	2012	TSCC	miR-200b and miR-15b	BMI1	CAL27, SCC25, CAL27-res and SCC25-res cell lines	Increased CDDP sensitivity
Yang (174)	2020	NPC	miR-200c	c-myc	149 patients CNE1, CNE2, 5–8 F cell lines	Increased CDDP sensitivity
Yang (178)	2021	HNC	miR-136-5p	ROCK1	FaDu and FD-LSC-1 cell lines	Increased CDDP sensitivity
Zhang (181)	2018	TSCC	miR-211-5p	Ezrin	102 patients CAL27, SCC9, CAL27-res and SCC9-res cell lines	Increased CDDP sensitivity.
Cao (182)	2020	NPC	miR-218-5p	GDPD5	CNE1, SUNE1, HNE1, and 5-8 F Cell lines	Increased CDDP sensitivity
Shan (186)	2015	TSCC	miR-338	HIF-1α	5 patients NP69, CNE2, CNE1, 5-8 F and 6-10B cell lines	Increased CDDP sensitivity
Yuan (188)	2021	NPC	miR-454	USP47	50 patients 5-8 F and SUNE-1 cell lines	Increased CDDP sensitivity
Song (190)	2021	OSCC	miR-619-5p	ATXN3	40 patients HOK, Leuk-1, HN4, HN6, CAL27, and UMSCC38 cell lines	Increased CDDP sensitivity
Yuan (195)	2017	NPC	miR-125b	Bcl-2 and MDR1	CNE2 cell line	Increased CDDP sensitivity
Gobin (196)	2023	LSCC	miR-9	ABCC1	UM-SCC-12 and UM-SCC-10 A cell lines	Increased CDDP sensitivity
Gao (197)	2022	OSCC	miR-188-3p	ABCB1	60 patients SCC-4, SCC-9, CAL-27, UM1, and UM2 cell lines	Increased CDDP sensitivity

## Role of miRNAs in pathology of head and neck cancers

Despite recent progresses in therapeutic methods, there is still a poor prognosis in advanced HNC. Regarding the heterogeneity of HNSCC, there is a need for the early diagnosis. Tumor metastasis is a complex process, including the tumor cells dissemination from the primary tumor, intravasation, extravasation, and secondary colonization. MiRNAs have a key role in tumor metastasis by regulation of EMT, invasion, and tumor cells self-renewal. EMT enhances tumor cell invasion, anoikis resistance, CSC features, and drug resistance in HNC tumors [36, 37]. MiRNAs have both oncogenic and tumor suppressor roles during HNC progression. OncomiRs are associated with malignant transformation and metastasis via the regulation of cell migration, proliferation, and angiogenesis [38, 39]. MiRNAs not only target a single gene, but they can also regulate an entire signaling pathway that shows the complexity of the intracellular interactions during the pathogenesis of LSCC [40]. Aberrant miRNAs expressions have been associated with chemo-radio resistance that introduce miRNAs as diagnostic and prognostic markers for HNC patients [41]. HNC cells with deregulation of miRNAs have different metastatic capabilities via EMT, CSC features, and aniokis. Therefore, evaluation of miRNAs in these processes uncovers the molecular mechanisms of HNC progression to introduce novel therapeutic and diagnostic markers for the HNC patients. Liquid biopsy is a key non-invasive diagnostic approach for the HNC cancers that can be done by the miRNA analysis in peripheral blood and saliva. This method improves the screening programs and early diagnosis for the real-time tumor monitoring in personalized medicine [42].

## Role of miRNAs in cDDP reponse in HNC cells by regulation of PI3K/AKT signaling pathway

**Fig. 1:**
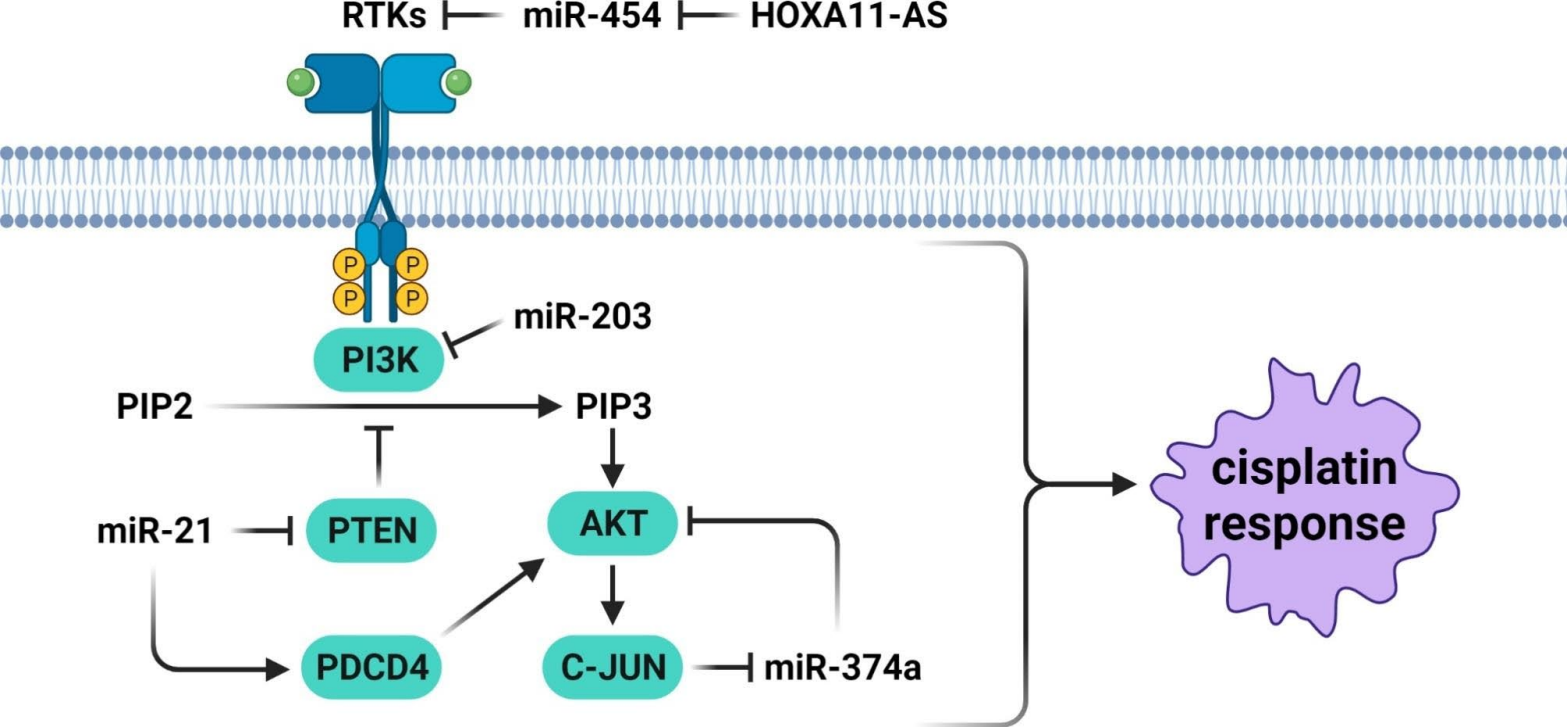
Role of miRNAs in CDDP response through the regulation of PI3K/AKT pathway in head and neck tumor cells. HOXA11-AS silencing promoted apoptosis and CDDP-sensitivity in NPC cells by suppressing the Met/Akt/mTOR pathway through up-regulation of miR-454-3p. MiR-21 enhanced cell proliferation and CDDP resistance through PTEN targeting in HNSCC cells. MiR-374a inhibited NPC cell growth and invasion, while enhanced CDDP sensitivity. PDCD4 up regulated the miR-374a via inhibition of PI3K/AKT/c-JUN signaling pathway. MiR-203 was associated with CDDP resistance through PIK3CA targeting in tongue squamous cancer. (Created with BioRender.com)

Phosphatidylinositol 3-kinase (PI3K) is a serine/threonine kinase that transfers the extracellular signals into the cells to mediate a variety of cellular processes. Protein kinase B (AKT) is the main effector of PI3K pathway that promotes cell growth and metabolism while inhibits the apoptosis [43]. Deregulation of PI3K/AKT is an important process that modulates multi-drug resistance (MDR) [44]. PIK3CA as the catalytic component of the PI3K complex is involved in tumor progression [45]. MiRNAs are involved in CDDP response in head and neck tumors by regulation of PI3K/AKT pathway (Fig. 1). It was found that miR-203 promoted cisplatin-induced apoptosis by inhibiting the self-renewal of cancer stem cells. MiR-203 was down regulated subsequent to the cisplatin treatment in tumor tissues. MiR-203 was associated with CDDP resistance through PIK3CA targeting in tongue squamous cancer [46]. C-Met as an activator of the PI3K/AKT pathway can induce mTOR and MDM2 while suppress BAD and GSK3 to promote cell growth and apoptosis resistance [47, 48]. Hence, the PI3K/AKT/mTOR axis and its downstream signaling cascade can promote tumor cell invasion and drug resistance through increasing cell cycle and inhibiting cell apoptosis [49, 50]. It has been investigated that HOXA11-AS silencing promoted apoptosis and CDDP-sensitivity in NPC cells by suppressing the Met/Akt/mTOR pathway through up-regulation of miR-454-3p [51]. IGF1R is a receptor tyrosine kinase that promotes the PI3K/AKT pathway. Circ_0005033 increased CDDP resistance in LSCC through miR-107/IGF1R axis [52]. PETN is an inhibitor of PI3K/AKT pathway. It was shown that miR-24 targeted PTEN/AKT pathway to promote cell viability and CDDP resistance in TSCC cells [53]. MiR-21 enhanced cell proliferation and CDDP resistance through PTEN targeting in HNSCC cells [54]. PDCD4 is a cytoplasmic tumor suppressor that inhibits PI3K/AKT/c-JUN pathway and cell cycle regulators including CCND1 and c-MYC that suppress cell cycle progression [55]. It is a tumor suppressor that inhibits tumor growth by interacting with eIF4A and eIF4G to suppress mRNA translation. It has been observed that miR-374a inhibited NPC cell growth and invasion, while enhanced CDDP sensitivity. MiR-374a was negatively associated with CCND1, PI3K/AKT, and c-JUN. CCND1 was the direct target of miR-347a and reduced the miR-374a mediated cell growth suppression, metastasis, and chemo resistance. C-JUN down regulated miR-374a and increased the levels of CCND1 expressions. PDCD4 up regulated the miR-374a via inhibition of PI3K/AKT/c-JUN signaling pathway [56]. MiR-21 promoted the CDDP resistance in OSCC cells by PTEN and PDCD4 targeting [57].

## Role of miRNAs in cDDP reponse in HNC cells by regulation of NOTCH, WNT, and Shh signaling pathways

**Fig. 2:**
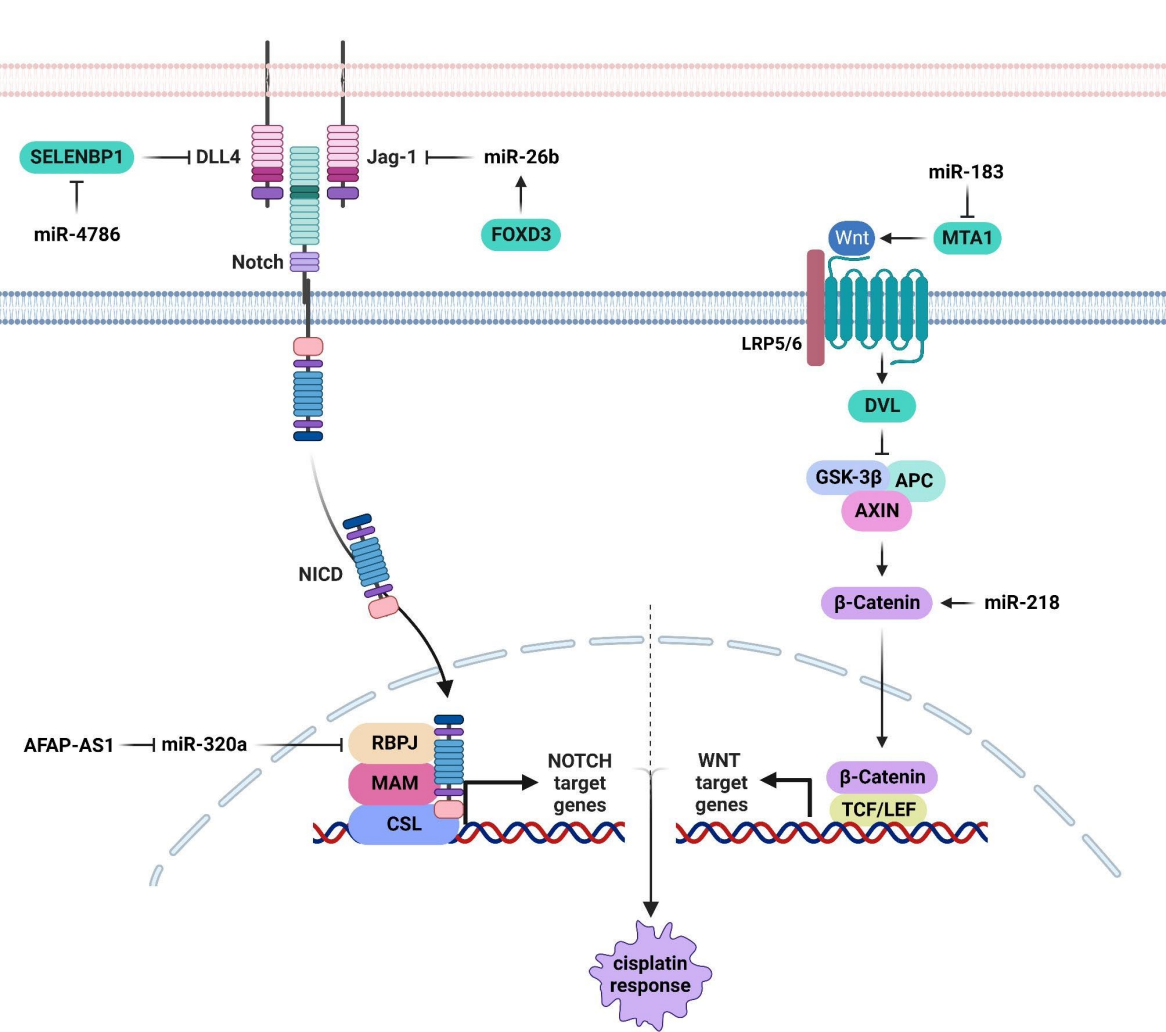
Role of miRNAs in CDDP response through the regulation of NOTCH and WNT pathways in head and neck tumor cells. FOXD3 regulated the miR-26b expression that subsequently targeted the JAG1 in NPC cells. AFAP1-AS1 increased laryngeal tumor cell chemo resistance and self-renewal via sponging miR-320a and RBPJ up regulation. MiR-183 increased CDDP sensitivity through MTA1 targeting in NPC cells. (Created with BioRender.com)

NOTCH is a developmental cell-cell adhesion dependent signaling pathway that is orchestrated by the ligand (JAG and DLL) binding to NOTCH receptors. Ligand binding induces the cleavage of NOTCH intracellular domain that enters to the nucleus where it regulates the expression of target genes by the CSL/MAML transcriptional complex [58]. NOTCH pathway has a crucial role in metastasis and chemo resistance of tumor cells [59, 60]. MiRNAs are involved in CDDP response of head and neck tumors by regulation of NOTCH pathway (Fig. 2). FOXC2 promoted chemo resistance of NPCs through EMT induction [61]. There was significant miR-26b down-regulation in CDDP resistant NPC compared with sensitive patients. FOXD3 regulated the miR-26b expression that subsequently targeted the JAG1 in NPC cells [62]. RBPJ is a pivotal transcriptional factor associated with NOTCH signaling [63, 64]. RBPJ binds to the NOTCH and serves as a transcriptional activator. RBPJ also suppresses gene expression in cooperation with co-repressors [65]. AFAP1-AS1 increased laryngeal tumor cell chemo resistance and self-renewal via sponging miR-320a and RBPJ up regulation [66]. SELENBP1 is a member of the selenium-binding protein family that is widely expressed in organs such as kidney, liver, heart, and lung [67]. SELENBP1 inhibits the malignant behaviors of cancer cells such as cell proliferation, migration, and EMT [68–70]. It suppresses tumor angiogenesis by binding and inhibiting the DLL4 in NOTCH pathway [71]. SELENBP1 was reported to inhibit chemo resistance in OSCC cells by functioning as a KEAP1 transcriptional activator, resulting in ubiquitination and degradation of NRF2. Down-regulation of SELENBP1 was associated with poor prognosis, increased tumor growth, and recurrence in OSCC patients. SELENBP1 down-regulation increased 5-FU and cisplatin resistance in OSCC cells. SELENBP1 targeting by miR-4786-3p promoted chemo-resistance in OSCC through modulation of KEAP1–NRF2 axis [72].

WNT is also a developmental signaling pathway involved in tumor progression and chemo resistance. It is orchestrated by binding of the WNT ligands to the FZD receptor that result in β-catenin activation. Then β-catenin enters into the nucleus to regulate WNT target genes by TCF/LEF transcriptional complex [73]. MiRNAs have critical roles in CDDP response of head and neck tumors by regulation of WNT pathway (Fig. 2). Protein Phosphatase 2 Regulatory Subunit B’Alpha (PPP2R5A) has been shown to regulate cell proliferation via the WNT pathway [74]. There was miR-218 up regulation in CDDP-resistant oral tumor cells and tissues. MiR-218 down regulation suppressed WNT signaling in oral tumor cells and promoted CDDP-mediated apoptosis through PPP2R5A targeting. MiR-218 up regulated the β-catenin and GSK3β while down regulated PPP2R5A [75]. Metastasis-associated protein 1 (MTA1) is a regulator of WNT1 signaling [76]. It has been shown that miR-183 increased CDDP sensitivity through MTA1 targeting in NPC cells [77]. Inhibition of miR-766-3p increased the CDDP sensitivity of HNSCC cells via NR3C2 targeting that induced β-catenin/c-Myc axis [78].

Sonic hedgehog (Shh) is a pivotal signaling pathway involved in cell proliferation and differentiation that functions by PTCH1 binding to the Hedgehog ligands and activation of smoothened (SMO) receptor following the SUFU release. Subsequently, the binding of PTCH1 to hedgehog activates GLI which in turn induces expression of GLI and PTCH1. GLI is involved in the transcriptional control of Sox2 and Nanog [79]. It has been shown that LINC-PINT decreased both cancer cell self-renewal and chemo resistance in laryngeal tumor cells. Deregulation of the LINC-PINT resulted in miR-425p up regulation and subsequent PTCH1 down regulation. Silencing of PTCH1 inhibited the GLI and its downstream targets such as Sox2 and Nanog [80]. Hyaluronan (HA) is a crucial extracellular matrix molecule in mammalians [81]. CD44 is a trans-membrane glycoprotein that is expressed in a wide variety of cells and tissues [82]. All of the CD44 isoforms have a HA binding site in their extracellular domain, rendering them a key HA cell surface receptor [83]. Nanog is a developmental transcription factor associated with self-renewal of stem cells [84]. It has been observed that the HA-CD44 interaction increased sphere formation, and self-renewal in CD44v3^high^ALDH1^high^ head and neck squamous cell tumor cells. HA-CD44v3 interaction also activated the Oct4, Sox2, and Nanog in these cells that along with miR-302 cluster activity were considered as the targets to overcome CDDP resistance in HNC tumors [85].

## Role of miRNAs in cDDP reponse in HNC cells by regulation of transforming growth factor β (TGF-β), NF-kb, and JNK signaling pathways

Transforming growth factor-β (TGF-β) is a pivotal intracellular signaling pathway in regulation of cell proliferation, cell adhesion, apoptosis, EMT, and drug resistance [86, 87]. TGFβ1 up-regulates the SOX4 during tumor progression [88–90]. SOX4 deregulation plays a pivotal role in cell cycle, apoptosis, chemo radiation response, and EMT [91–95]. It has been observed that TGFβ1 up regulated the SOX4 following the miR-34c down-regulation, which resulted in EMT induction and CDDP resistance in NPC cells [96]. MiR-132 decreased cell proliferation and invasion, while induced CDDP sensitivity in OSCC cells via TGF-β1 inhibition [97].

**Fig. 3:**
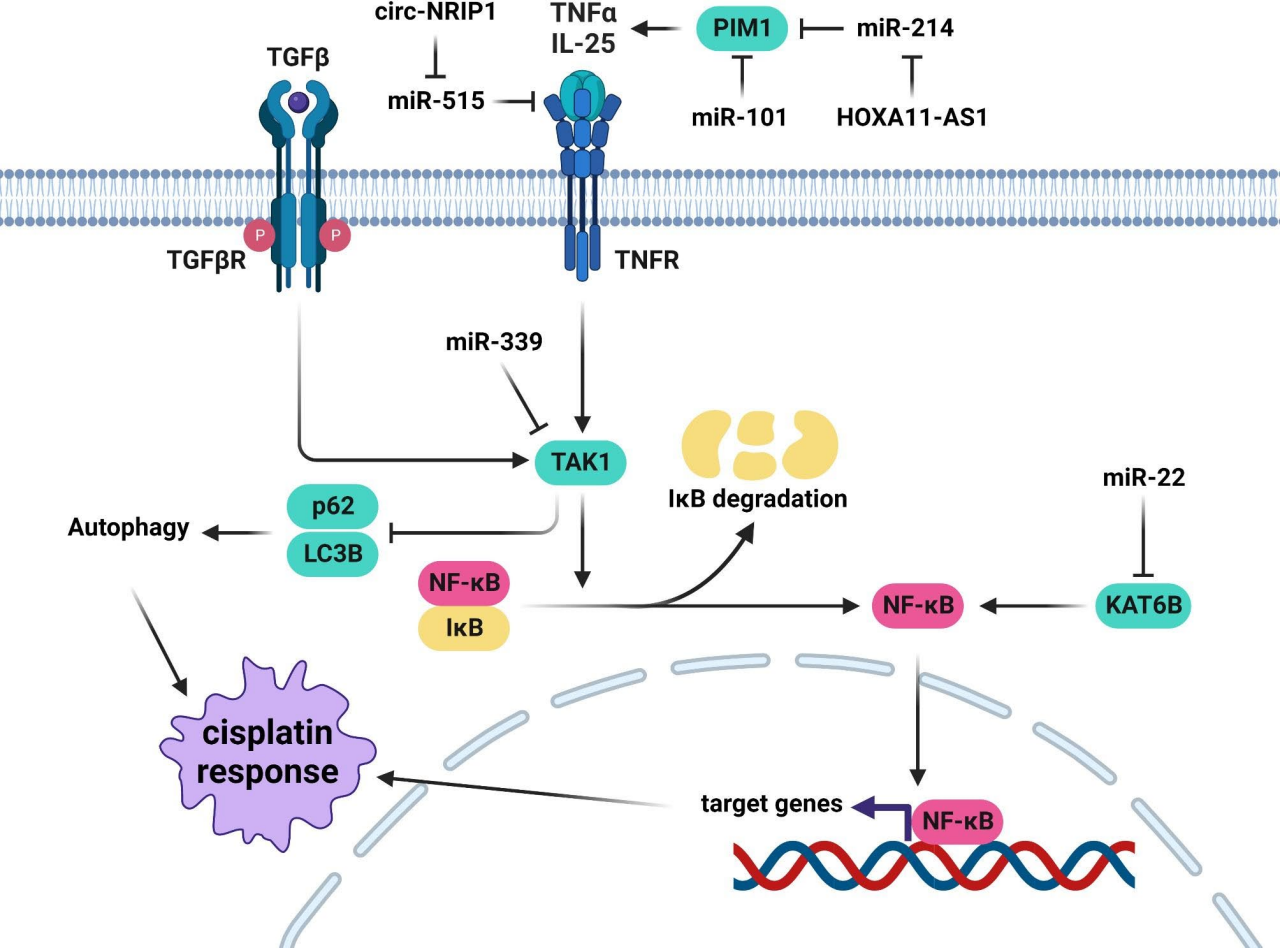
Role of miRNAs in CDDP response through the regulation of NF-kB pathway in head and neck tumor cells. Enhanced chemo sensitivity in tongue cancer cells could be achieved by miR-22-mediated down regulation of KAT6B. Circ-NRIP1 down regulation decreased the CDDP-resistance of NPC cells by regulating the miR-515-5p/IL-25 axis. MiR-101-3p promoted apoptosis and CDDP sensitivity in ACC cells through Pim1 targeting. HOXA11-AS promoted CDDP-resistance by modulating the miR-214-3p/PIM1 axis. MiR-339-5p suppressed autophagy to increase CDDP sensitivity in laryngeal carcinoma cells through TAK1 targeting. (Created with BioRender.com)

NF-κB is a key signaling pathway involved in tumor progression by regulation of cell proliferation and angiogenesis [98]. MiRNAs are the key players in CDDP response of head and neck tumors by regulation of NF-κB pathway (Fig. 3). KAT6B is a Histone acetyltransferase involved in regulation of cell cycle, DNA repair, and signal transduction [99]. There was a significant correlation between miR-22 expression and CDDP sensitivity in tongue cancer patients. It was suggested that the enhanced chemo sensitivity in tongue cancer cells could be achieved by miR-22-mediated down regulation of KAT6B, which results in decreased NF-kB activity and increased cell death in response to chemotherapy [100]. IL-25 has been shown to promote chemo resistance in tumor cells by activating the NF-κB signaling [101]. There was a significant circ-NRIP1 up-regulation in the serum samples of CDDP-resistant NPC compared with sensitive patients. Circ-NRIP1 down regulation decreased the CDDP-resistance of NPC cells by regulating the miR-515-5p/IL-25 axis [102]. Adenoid cystic carcinoma (ACC) is an uncommon neoplasm of salivary glands with neural and vessel invasion and poor long-term survival rate due to a high risk of distant metastasis [103, 104]. A third of ACC patients with distant metastasis are likely to die within two years [105]. Tumor cells commonly acquire MDR subsequent to the administration of a single chemotherapy drug, which accounts for the majority of cancer-related mortality [106]. PIM1 belongs to the active serine/threonine kinase family that promotes tumor progression by regulation of cell cycle, cell death, and signaling pathways [107]. It activates NF-kB signaling following the TNF-α induction by RelA/p65 recruitment [108]. There was a significant miR-101-3p down regulation in ACC tissues compared with normal parotid glands. MiR-101-3p inhibited cell proliferation, invasion, and colony formation, while promoted apoptosis and CDDP sensitivity in ACC cells through Pim-1 targeting [109]. There was HOXA11-AS up-regulation in CDDP-resistant OSCC cells. It promoted CDDP-resistance by modulating the miR-214-3p/PIM1 axis [110].

C-Jun N-terminal kinases (JNKs) are the members of the MAP kinases that are involved in stress response, cell death, and senescence [111]. ATF2 is a cAMP response element binding family member which is activated by JNK [112, 113]. DNA damage causes ATF2 phosphorylation via activating the JNK pathway. Phosphorylated ATF2 subsequently promotes DNA repair via targeting the factors involved in cell survival. Therefore, phosphorylation of ATF2 results in CDDP resistance through promoting the DNA-repair [114, 115]. MiR-26b reduced CDDP resistance through ATF2 targeting in laryngeal tumor cells [116].

## Role of miRNAs in cDDP reponse in HNC cells by regulation of apoptosis and autophagy

**Fig. 4:**
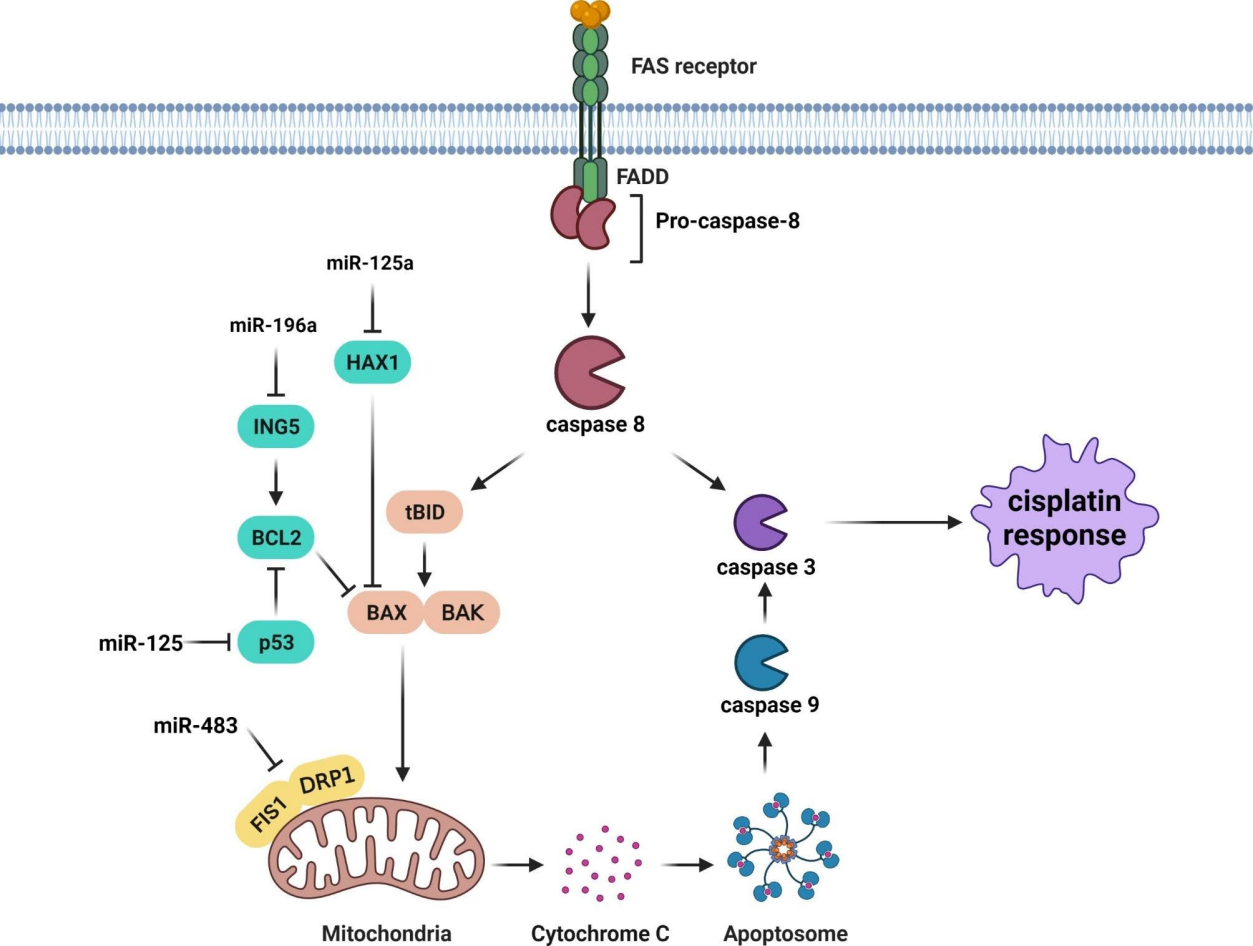
Role of miRNAs in regulation of CDDP mediated apoptosis in head and neck tumor cells. MiR-483-5p suppressed mitochondrial fission and CDDP sensitivity via FIS1 targeting in TSCC cells. MiR-196a modulated cell proliferation and apoptosis via targeting ING5. There were miR-125 up regulations in CDDP-resistant NPC tissues in comparison with normal tissues which were associated with p53 targeting. MiR-125a promoted CDDP-induced apoptosis through HAX1 targeting in laryngeal cancer stem cells. (Created with BioRender.com)

CDDP forms DNA adducts that can be finally results in apoptosis induction. Therefore, miRNAs can also affect the CDDP response by regulation of apoptosis pathway in head and neck tumors (Fig. 4). Mitochondrial fission triggers the apoptosis by the release of pro-apoptotic factors that activate caspase proteins. Abnormal mitochondrial dynamics are implicated in the regulation of apoptosis and have been associated with a variety of disorders [117–119]. Different proteins, such as DRP1, FIS1, and MFF in mammalian cells, mediate mitochondrial fission. DRP1 is required for mitochondrial fission, and its inhibition leads to decreased cell death in various tumors [120]. FIS1 acts as a DRP1 receptor, allowing DRP1 to enter mitochondria and carry out mitochondrial fission and cell death [121, 122]. It has been reported that miR-483-5p suppressed mitochondrial fission and CDDP sensitivity via FIS1 targeting in tongue squamous cell carcinoma (TSCC) cells [123]. The importance of the tumor microenvironment in tumor development and chemo resistance has been the subject of numerous studies [124]. Cancer-associated fibroblasts (CAFs) have a pivotal role in HNC progression through extracellular matrix remodeling, growth factors secretion, and therapeutic resistance induction [124–126]. ING5 proteins regulate the expression of many genes, including p53 target genes BAX and p21 [127]. It was observed that HNC-derived CAFs were intrinsically resistant to cisplatin and that CAF-CM could promote HNC cell proliferation and survival following cisplatin administration. CAF-derived exosomes carrying miR-196a were shown to be associated with cisplatin resistance in HNC cells. MiR-196a increased cell proliferation, while decreased cell death in HNC subsequent to cisplatin therapy. After being transported from CAFs to HNC cells, exosomal miR-196a modulated cell proliferation and apoptosis via targeting CDKN1B and ING5. MiR-196a up-regulation was also associated with CDDP resistance in HNC cells [128]. There was GAS5 down regulation in CDDP-resistant OSCC cells and tissues that were correlated with survival rates. GAS5 recovered the CDDP sensitivity in OSCC cells by miR-196a sponging [129]. There were miR-125 up regulations in CDDP-resistant NPC tissues in comparison with normal tissues which were associated with p53 targeting [130]. SF1 RNA-binding protein is involved in the formation of spliceosomes [131]. There was a significant UCA1 up regulation in OSCC tissues compared with normal samples. UCA1 increased OSCC cells proliferation and CDDP-resistance through miR-184 sponging and SF1 up regulation. UCA1 down-regulation could be associated with BAX up regulation, caspase-3 activity, and BCL2 inhibition [132]. Zinc finger antisense 1 (ZFAS1) is involved in regulation of cell death, cell proliferation, and invasion [133]. ZFAS1 up regulation was correlated with increased cell proliferation and CDDP resistance via miR-421 sponging and MEIS1 up regulation in OSCC cells. ZFAS1 had an anti-apoptotic effect via modulating CASP-3, BAX, and BCL2 expression in CDDP resistant OSCC cells [134]. HAX-1 suppresses the mitochondrial apoptosis pathway by reducing the accumulation of BAX. Hence, HAX-1 protects cancer cells against drug-induced apoptosis [135–137]. MiR-125a promoted CDDP-induced apoptosis through HAX-1 targeting in laryngeal cancer stem cells [138]. GPR78 is a G-protein coupled receptor that serves as a death receptor to activate cell death [139]. There was miR-936 down regulation in Laryngeal Squamous Cell Carcinoma (LSCC) compared with normal tissues that was associated with poor clinical outcomes. MiR-936 suppressed LSCC cell proliferation, migration, and CDDP resistance by GPR78 targeting [140].

Autophagy is a cell survival mechanism that includes self-degradation through the transportation of cytoplasmic ligands to the lysosome to maintain body equilibrium due stressful conditions such as hypoxia and nutritional deficiency [141]. Autophagy has been shown to significantly reduce the accumulation of damaged proteins and organelles, thereby inhibiting tumorigenesis [142]. It was reported that autophagy was commonly induced in tumor cells through both chemotherapy and radiation, preserving the tumor cells from the antitumor therapy [143]. Drug resistance and tumor growth have been reported to be frequently promoted through the autophagic flux in advanced malignancies via ensuring tumor cell survival by maintaining essential energy production [144, 145]. High mobility group box-1 protein (HMGB1) is involved in DNA repair, cell proliferation, and apoptosis [146]. HMGB1 was also associated with therapeutic tolerance in some cancers as a key autophagy regulator [147]. H19 up-regulation was correlated with autophagy-mediated drug in LSCC. H19 increased CDDP sensitivity by miR-107/HMGB1 targeting in LSCC [148]. There was a significant miR-1278 down regulation in NPC tissues that was correlated with poor survival and chemotherapy response. MiR-1278 increased DDP sensitivity in NPC cells and decreased autophagy through ATG2B targeting [149]. It was found that circPGAM1-mediated drug resistance was associated with miR-376a in laryngocarcinoma. CircPGAM1 enhanced CDDP resistance by miR-376a/ATG2A targeting in laryngocarcinoma [150]. Circ-PKD2 induced Atg13-mediated autophagy through miR-646 sponging to promote the CDDP sensitivity in oral squamous cell carcinomas [151]. The increased LC3-II/LC3-I ratio is commonly linked to autophagy [152]. TAK1 is an important key regulator of signal transduction, that has an essential role in TGF-β-mediated EMT and apoptosis through controlling the JNK and p38 pathway [153]. TAK1 is also able to enhance tumor cell proliferation, metastasis, and invasion [154]. MiR-339-5p suppressed autophagy to increase CDDP sensitivity in laryngeal carcinoma cells through TAK1 targeting. MiR-339-5p could efficiently reduce the LC3-II/LC3-I ratio in CDDP-resistant laryngeal carcinoma cells [35]. There was a significant miR-205 down-regulation in nasopharyngeal carcinoma cells. MiR-205 inhibited the cell proliferation and invasion, while enhanced CDDP sensitivity in CNE1 cells. MiR-205 also increased autophagy through LC3B II up-regulation and p62 down-regulation in the nasopharyngeal carcinoma cells [155].

## Role of miRNAs in cDDP reponse in HNC cells by regulation of epithelial-mesenchymal transition

Epithelial to mesenchymal transition (EMT) is a cellular mechanism in which epithelial cells obtain the mesenchymal phenotype via down regulation of the epithelial markers like CDH1, as well as up-regulation of mesenchymal markers like Vimentin and CDH2 [156–158]. EMT is defined as the loss of polarity, cell-cell adhesion, and cytoskeletal components in epithelial cell layers [159]. There was a considerable miR-205-5p up-regulation in the CDDP-resistant cells in comparison with the parental cells. MMP-2 and MMP-9 were down regulated via miR-205-5p in HNE1 cells. MiR-205-5p down regulated CDH1 while up regulated the Vimentin and, CDH2, Slug, and SNAI1 in HNE1 cells. MiR-205-5p enhanced the EMT through PTEN inhibition in CDDP-resistant NPC cells [160]. Twist1 is also an EMT specific transcription factor that can be activated by different signaling pathways [161]. It has been shown that miR-23a induced CDDP resistance via Twist1 targeting in TSCC cells [162]. Yes Associated Protein (YAP) is the main nuclear effector in Hippo pathway that is involved in tumor growth. MiR-15b induced mesenchymal-epithelial transition and CDDP sensitivity in SCC25 cells by TRIM14 targeting. TRIM14 significantly up regulated YAP in the SCC25 cells [163]. PBX3 is a transcription factor involved in EMT process and tumor progression [164–166]. Inhibition of HOXA11AS promoted the CDDP sensitivity of NPC cells via miR-98/PBX3 axis [167].

BMI1 belongs to the polycomb proteins and suppresses CDH1 transcription via PRC1/PRC2- related chromatin remodeling to promote EMT [168]. It can stabilize Snail via the regulation of PI3K/AKT/GSK-3β pathway [169]. MiR-200b and miR-15b down regulations were implicated in chemotherapy-induced EMT and chemo-resistance in TSCC cells. They reversed mesenchymal characteristics and inhibit tumor invasion in chemo resistant TSCC cells through BMI1 targeting. Down regulation of miR-200b and miR-15b were also contributed with lymph node involvement [170]. C-Myc transcription factor has been shown to regulate cell proliferation, apoptosis, metabolism, and genomic stability [171–173]. C-Myc expression was associated with miR-200c suppression through directly binding to the miR-200c promoter in primary NPC tumors. MiR-200c down regulated the CDH1 and up regulated Vimentin through ZEB2 targeting. BMI1, Suz12, and Sox2 were also down regulated by miR-200c or c-Myc suppression. Therefore, C-Myc/miR-200c axis was found to be a negative regulatory feedback loop that has a pivotal role in the EMT, chemotherapy resistance, and CSC phenotypes in nasopharyngeal cancer [174].

ROCK is an effector of Rho A that interacts with actin cytoskeleton to induce the generation of focal adhesion and tumor cell invasion [175, 176]. It is also involved in regulation of cell proliferation and migration, and EMT. It plays a critical role in TGF-induced EMT by activating RhoA-dependent pathways [177]. MiR-136-5p suppressed LSCC and HPSCC cells migration while promoted CDDP sensitivity via ROCK1 targeting. It up regulated CDH1 and down regulated the CDH2 and vimentin. Over expression of miR-136-5p in combination with CDDP down regulated the p62 and suppressed the Akt/mTOR pathway [178]. EZR is a linkage protein between the membrane proteins and actin cytoskeleton [179]. It has also a significant role in chemo-resistance [180]. KCNQ1OT1 up regulation in CDDP resistant TSCC samples were indicated to be associated with poor prognosis. KCNQ1OT1 promoted cell proliferation and CDDP resistance through controlling the EZR/FAK/SRC axis via miR-211-5p [181]. Increased levels of MAGI2-AS3 were reported to be associated with enhanced cell proliferation, migration, and EMT by miR-218-5p/GDPD5/SEC61A1 axis in NPC. MAGI2-AS3 increased CDDP resistance through GDPD5 regulation [182].

Hypoxia has been identified as a characteristic of the variety of malignant tumor microenvironments [183, 184]. Hypoxia promotes tumor cells invasiveness and chemo resistance and leads to the poor clinical outcomes. Hypoxia-inducible factor 1-alpha (HIF-1a) is the crucial regulator of angiogenesis and hypoxia that is used as a prognostic marker in NPC [185]. It has been found that down regulation of miR-338-3p suppressed tumor proliferation via HIF-1α targeting. MiR-338-3p inhibited the CNE2 cell proliferation and migration and reversed hypoxia-induced CDDP resistance and EMT [186]. USP47 is an ubiquitin peptidase involved in hypoxia-induced EMT by SNAI1 deubiquitination and stabilization [187]. KCNQ1OT1 knockdown significantly inhibited NPC cell viability, while induced CDDP sensitivity via miR-454/USP47 axis [188]. ATXN3 is a deubiquitinase involved in cell homeostasis and tumor progression. It promoted the lung cancer through KLF4 deubiquitinating [189]. It has been found that miR-619-5p reduced OSCC cells migration via PI3K/AKT pathway. MiR-619-5p increased CDDP sensitivity in OSCC cells by ATXN3 targeting [190].

## Role of miRNAs in cDDP reponse in HNC cells by regulation of transporters

MDR is a significant challenge during the cancer treatment that is mainly associated with the efflux of drugs through the ATP-binding cassette (ABC) transporters [191]. MDR1 belongs to the ABC transporter protein family that confer MDR by keeping the intracellular concentration of hydrophobic chemicals below a cell-killing threshold through an active transport mechanism [192]. MRP1 is also an ABC transporter, promoting MDR in tumor cells through reduced anticancer medication absorption [193]. It was observed that the up-regulation of circ_0004507 was associated with tumor stage, lymph node metastasis, and CDDP resistance in laryngeal cancer tissues. MRP1 and MDR1 protein levels were reduced subsequent to the Circ_0004507 down-regulation. Circ_0004507 enhanced the tumor progression and CDDP resistance of laryngeal cancer cells through miR-873 sponging [194]. There was a significant miR-125b down regulation in CNE2/DDP resistant cells. MiR-125b increased apoptosis and CDDP-sensitivity of tumor cells via Bcl-2 and MDR1 targeting [195]. MiR-9 reduced cell proliferation and migration while promoted the CDDP sensitivity via ABCC1 targeting in laryngeal tumor cells [196]. Circ_0109291 induced CDDP resistance by regulation of miR-188-3p/ABCB1 axis in of OSCC cells [197].

## Conclusions

Head and neck tumors are recognized as a global health challenge due to their digestive and nutritional problems for the patients. Although, these tumors have a high chance of treatment and good prognosis, a significant proportion of these tumors are diagnosed in the advanced stages with a poor prognosis. CDDP as a first-line treatment has a critical role in the treatment of head and neck cancer patients. However, CDDP resistance can be observed in a significant rate of patients. We investigated the role of miRNAs in CDDP response of head and neck cancers. It has been reported that miRNAs affect the CDDP response in head and neck tumors by regulating signaling pathways, autophagy, apoptosis, and membrane transporters. Since, miRNAs has a higher stability in body fluids in comparison with the mRNAs; they can be suggested as non-invasive markers to predict the CDDP response in head and neck tumors. Therefore, CDDP response prediction by miRNA based panel markers can reduce the CDDP side effects and helps to define the most efficient therapeutic modality based on the personalized medicine for these cancer patients. MiRNA-based therapy can be associated with the miRNA function by the promotion of tumor suppressor miRNAs (mimics) while suppression of oncogenic miRNAs (antagomiRs) in tumor cells. Therefore, antagomiRs or mimics can be used to overcome the CDDP resistance in HNC patients. However, the cytoplasmic miRNA degradation and their side effects in normal tissues are the main therapeutic challenges in miRNA-based treatments. Therefore, the site-specific miRNA delivery can reduce the concentrations of antagomiRs or mimics that reduce the probable side effects in normal tissues.

## Data Availability

Not applicable.

## List of Abbreviations

CDDP: Cisplatin
miRNAs: MicroRNAs
HNCs: Head and neck cancers
HNSCC: Head and neck squamous cell carcinoma
5-FU: 5-fluorouracil
NPC: Nasopharyngeal carcinoma
OSCC: Oral squamous cell carcinoma
PI3K: Phosphatidylinositol 3-kinase
MDR: Multi-drug resistance
PPP2R5A: Protein Phosphatase 2 Regulatory Subunit B’Alpha
MTA1: Metastasis-associated protein 1
Shh: Sonic hedgehog
SMO: Smoothened
HA: Hyaluronan
TGFβ: Transforming growth factorβ
ACC: Adenoid cystic carcinoma
JNKs: C-Jun N-terminal kinases
CAFs: Cancer-associated fibroblasts
OSCC: Oral squamous cell carcinoma
ZFAS1: Zinc finger antisense 1
LSCC: Laryngeal Squamous Cell Carcinoma
HMGB1: High mobility group box-1 protein
EMT: Epithelial to mesenchymal transition
YAP: Yes Associated Protein
HIF-1a: Hypoxia-inducible factor 1-alpha
ABC: ATP-binding cassette
